# Systematic review and meta-analysis comparing surgical site infection in abdominal surgery between triclosan-coated and uncoated sutures

**DOI:** 10.1007/s10029-024-03045-5

**Published:** 2024-05-07

**Authors:** Martijn Depuydt, Sarah Van Egmond, Stine Mette Petersen, Filip Muysoms, Nadia Henriksen, Eva Deerenberg

**Affiliations:** 1 General Surgery, AZ Maria Middelares, Buitenring-Sint-Denijs 30, 9000 Ghent, Belgium; 2 Department of Surgery, University of Ghent, Ghent, Belgium; 3 Department of Surgery, Franciscus Gasthuis and Vlietland, Kleiweg 500, 3045 PM Rotterdam, The Netherlands; 4 Department of Surgery, Erasmus University Medical Center, Rotterdam, The Netherlands; 5 Hepatic and Digestive Diseases, Herlev University Hospital, Copenhagen, Denmark

**Keywords:** Surgical site infection, Triclosan, PDS plus, Vicryl plus, Fascial closure, Wound closure, Hernia prevention, Surgical complication

## Abstract

**Purpose:**

Surgical site infection (SSI) is a frequent complication after abdominal surgery and impacts morbidity, mortality and medical costs. This systematic review evaluates whether the use of triclosan-coated sutures for closing the fascia during abdominal surgery reduces the rate of SSI compared to uncoated sutures.

**Methods:**

A systematic review and meta-analysis were conducted using the PRISMA guidelines. On February 17, 2024, a literature search was performed in Medline ALL, Web of Science Core Collection, Cochrane Central Register of Controlled Trials and Embase. Randomized controlled trials (RCTs) on abdominal fascial closure in human adults, comparing triclosan-coated and uncoated sutures, were included. The risk of bias was assessed using the Cochrane RoB 2 tool. Pooled meta-analysis was performed using RevMan.

**Results:**

Out of 1523 records, eleven RCTs were included, with a total of 10,234 patients: 5159 in the triclosan-coated group and 5075 in the uncoated group. The incidence of SSI was statistically significantly lower in the triclosan-coated group (14.8% vs. 17.3%) with an odds ratio (OR) of 0.84 (95% CI [0.75, 0.93], *p* = 0.001). When polydioxanone was evaluated separately (coated *N* = 3999, uncoated *N* = 3900), triclosan-coating reduced SSI; 17.5% vs. 20.1%, OR 0.86 (95% CI [0.77; 0.96], *p* = 0.008). When polyglactin 910 was evaluated (coated *N* = 1160, uncoated *N* = 1175), triclosan-coating reduced the incidence of SSI; 5.4% vs. 7.8%, OR 0.67 (95% CI [0.48; 0.94], *p* = 0.02).

**Conclusion:**

According to the results of this meta-analysis the use of triclosan-coated sutures for fascial closure statistically significantly reduces the incidence of SSI after abdominal surgery with a risk difference of about 2%.

**Supplementary Information:**

The online version contains supplementary material available at 10.1007/s10029-024-03045-5.

## Introduction

Surgical site infection (SSI) is an infection occurring within thirty days after a surgical procedure involving the surgical area [1]. It is one of the most common complications after abdominal surgery and occurs in 3–20% of patients [2]. SSIs may cause pain, increase the use of antibiotics, prolong the length of hospital stay, increase the risk of readmission, diminish wound healing and thereby increase the long-term risk of incisional hernia [3, 4]. SSIs have a substantial financial impact and can increase hospital costs by $21-$34,000 per SSI [5]. Prevention of SSIs is therefore key. Disinfection, hand sanitation, rules of sterility, prophylactic antibiotics, prevention of blood loss, shorter operation times and other aseptic protocols are factors that are well-known to reduce the risk of SSI [1, 6, 7].

At the beginning of this century, triclosan-coated sutures were developed to further reduce SSI. By damaging the cytoplasmic membrane of both gram-negative and gram-positive bacteria, triclosan has bactericidal properties [8, 9]. The triclosan-coating lasts one month and may lead to a decrease in late infection [10]. Triclosan has been safely used for many years in products such as toothpaste and soap. However, triclosan may lead to bacterial resistance and contribute to reduced susceptibility to clinically important antimicrobials, due to either cross-resistance or co-resistance [9]. Other possible disadvantages of triclosan include allergic dermatitis and toxicity [11]. The use of Triclosan is sometimes controversial because, based on GreenScreen^®^ hazard assessment, it is a product of serious concern that needs to be avoided. This is mostly due to toxicity to nature and to humans when inhaled and when daily used. The most important hazard concerning triclosan coating of sutures is a Globally Harmonized System (GHS) category of two for skin irritation, which means it can cause reversible change to skin if used longer than four hours. [12] Preclinical, phase 0 and phase 1 studies have shown that the systemic concentration of triclosan is low and safe when triclosan-coated sutures are used [13].

Early wound complications are a risk factor for the development of incisional hernias [14]. The guidelines of European and American Hernia Societies on the closure of abdominal wall incisions do not recommend the use of triclosan-coated sutures due to insufficient data [15]. In 2017, a systematic review comparing coated and uncoated sutures showed a statistically significant benefit in favour of triclosan-coating on fast-absorbable braided polyglactin 910, but this difference was not noticed when considering slowly absorbable monofilament polydioxanone [16]. New randomized controlled trials (RCTs) have since been published, which led to an update of this review. Our hypothesis is that the triclosan-coating of the sutures used in the closure of the abdominal fascia, can influence the incidence of SSI. The aim of this study is to perform a systematic review and meta-analysis of RCTs comparing the effect of triclosan-coated and uncoated sutures on the incidence of SSI.

## Materials and methods

On February 17, 2024, a literature search was performed in Medline ALL, Web of Science Core Collection, Cochrane Central Register of Controlled Trials and Embase. The search terms, inclusion criteria and exclusion criteria can be found in Table 1. The search string was built and adjusted by the medical library of the Erasmus University Medical Center using the (population, intervention, comparison, outcomes and study) PICO-criteria, composed of Emtree and Medical Subject Headings (MeSH)-terms. A detailed description of the search string can be requested from the corresponding author. The study protocol of this systematic review was registered in the PROSPERO database prior to the search with the following identification number: CRD42022332317.

**Table 1:** Search terms, inclusion and exclusion criteria

Search terms	Inclusion criteria	Exclusion criteria
Laparotomy Laparoscopy Suture Triclosan Polydioxanone Polyglactin 910 Surgical site infection Wound infection Controlled clinical trial Synonyms of the terms above	Adults (≥ 18 years) Randomized controlled trial Comparing triclosan-coated sutures versus uncoated sutures Reporting superficial and deep SSI Appropriate and prearranged definition for SSI Laparotomy/laparoscopy Emergency/elective surgery Clean/clean-contaminated/contaminated/infected surgical procedures All languages	Children (< 18 years) Animal studies High risk of bias according to the Cochrane RoB 2 Tool No full-text available Cohort studies Study protocols Observational studies Organ/space infections

The objective of this study was to conduct an updated literature search and evaluate the results of RCTs comparing triclosan-coated and uncoated sutures. The primary outcome was SSI incidence. A first-level screening was performed on every part by two different members of the study team based on the title and abstract. All the teams consisted one graduated surgeon and expert in the field and one resident or PhD candidate in the surgical field. A second-level screening based on the full-texts was performed by a different pair of the study team. RCTs on abdominal fascial closure in adults (≥ 18 years) comparing triclosan-coated and uncoated sutures in the elective or emergency settings were included. The type of surgery was open or laparoscopic abdominal surgery. Both superficial and deep SSIs according to the Centers for Disease Control and Prevention (CDC) criteria were both included as primary outcome. Organ or space infections were not included. An appropriate definition for SSI was required for inclusion. Articles in every language were included, most could be understood by the international study team. When this was not the case, the artificial intelligence-powered translation software Pairaphrase^®^ [17] was used, controlled by the second software Google translate^®^ [18]. Studies on animals, paediatric surgery or non-abdominal surgery were excluded. Different institutional electronic and physical libraries were consulted. When no full-text was available, electronic contact was sought with the research group. If no contact could be made, the article was excluded. Duplicates were removed.

The effects of the triclosan-coating on polydioxanone and polyglactin 901 were analyzed separately. Furthermore, we performed a subgroup analysis considering the effect of triclosan-coated sutures excluding low- and middle-income countries, defined according to the World Bank income classification, as countries with a Gross National Income per capita less than $13,845 [19]. This subanalysis was not a planned “a priori” subgroup analysis but was performed because with an SSI rate of 21.5% in low- and middle-income countries and 9.7% in high-income countries, there is a remarkably higher rate of SSI in the low- and middle-income countries.

The risk of bias was checked by at least two different researchers with the Risk of Bias 2 (RoB 2) tool [20]. When two research members could not agree, a third member of the study team was consulted to reach consensus. Articles that scored ''high risk of bias'' based on the RoB 2 tool were excluded. The data were extracted by one research member and controlled by a second research member.

A fixed-effects model was used to pool the data. The outcome SSI was dichotomous and the odds ratio (OR) was calculated with the Mantel–Haenszel method with a 95% confidence interval. The risk of type I and II errors was set to 5% and 20%, respectively. When heterogeneity was assessed, Chi2 tests with *p* < 0.05 and I2 > 50% were considered to indicate significant heterogeneity. The data were analyzed with Review Manager 5 [21]. A sensitivity analysis was performed for all the results. A funnel plot was constructed to detect publication bias. The results of the systematic review and meta-analysis are reported in line with the guidelines of PRISMA [22]. The quality of this review was optimized using the MOOSE checklist [23] and the AMSTAR checklist [24].

## Results

**Fig. 1:**
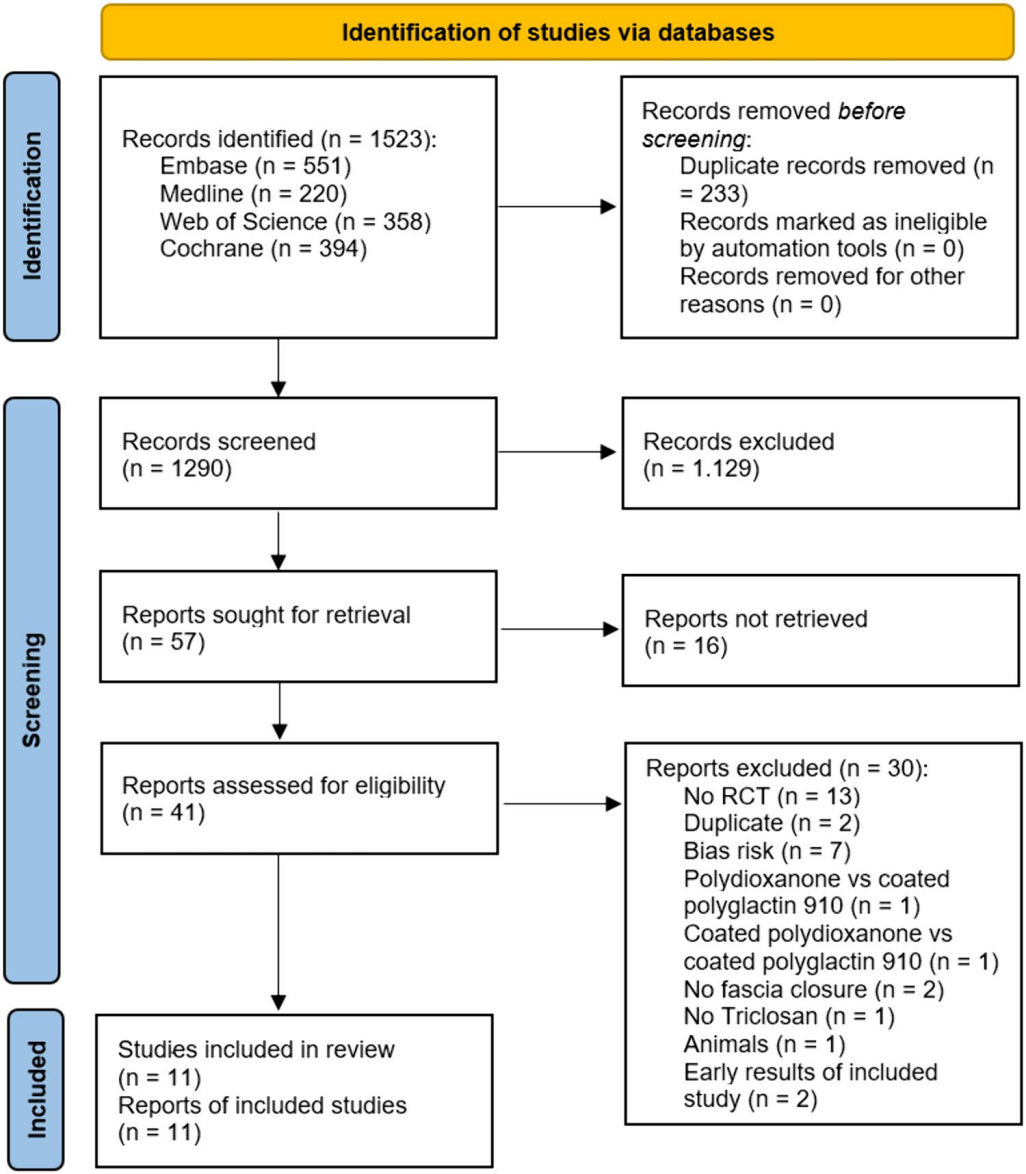
PRISMA for studies concerning SSI prevention after abdominal incisions with triclosan-coated sutures

**Fig. 2:**
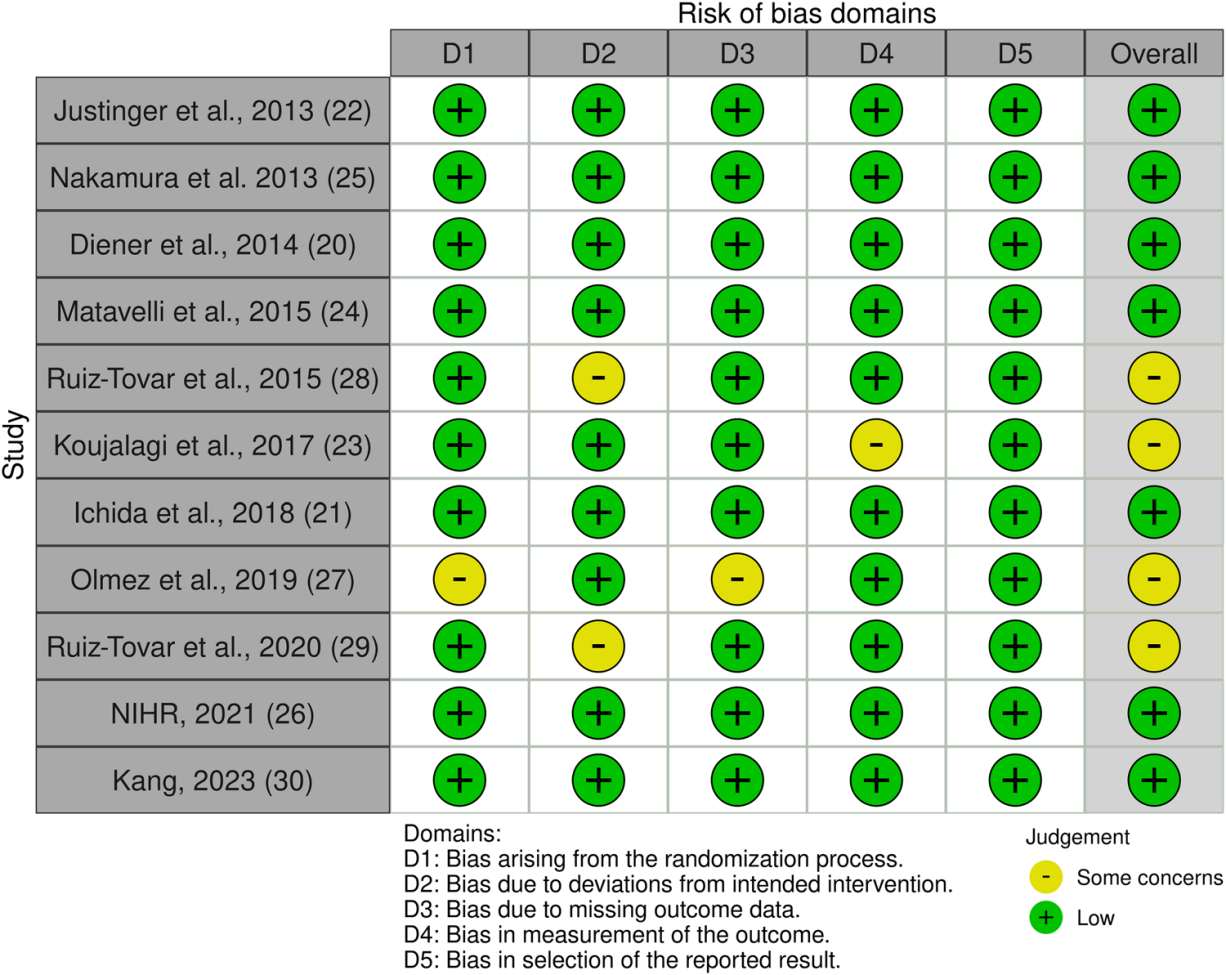
Risk of bias table of the included studies, created with RevMan

Out of 1523 records, 41 full-text articles were assessed for eligibility. Thirty full-text articles were assessed and excluded from the analysis, seven of which were excluded due to a high risk of bias. The considerations for exclusion can be found in the PRISMA-flowchart, shown in Fig. 1. A list of excluded articles can be requested from the corresponding author. Eleven RCTs could be included in the meta-analysis [25–35]. According to the RoB 2 tool [20], the risk of bias was low in seven and of some concerns in four of the included studies. An overview is presented in Fig. 2.

A total of 10,234 patients were included: 5159 in the triclosan-coated suture group and 5075 in the uncoated suture group. The study characteristics of the included studies can be found in Table 2. Patient demographic similarity was reported in all studies. The SSI incidence in the triclosan-coated suture group ranged from 4.4 to 30% and in the uncoated suture group from 5.1 to 43.3%. All but one [32] study used the CDC criteria for diagnosing SSI [1]. That study of Olmez et al. [32] did use similar criteria: clinical signs of SSI by physician designee with or without deliberate opening of the wound or purulent drainage. An aseptic specimen was obtained when purulent drainage was present, within thirty days after surgery. Five studies [25, 26, 29, 32, 35] reported superficial and deep SSIs separately. Organ and space SSIs were not included. Seven studies with a total of 7899 patients [10, 25, 27, 28, 31, 32, 34] used polydioxanone sutures. Four studies with a total of 2335 patients [26, 30, 33, 35] used polyglactin 910 sutures. In the RCT of Ruiz-Tovar et al. [34], patients were randomized into three groups: triclosan-coated barbed suture, triclosan-coated polydioxanone suture and uncoated polydioxanone suture. The data of the last two groups were extracted. The groups were separately randomized and the group demographics were statistically similar among the three groups. This was the reason the risk of bias in this study was of ‘some concerns’.

**Table 2:** Study characteristics of the included studies

Study	Type of study	Number of patient (Triclosan-coated/uncoated)	Surgery characteristics	Inter-vention	Number of SSI events (%) in intervention group	Comparison	Number of SSI events (%) in control group	Method of evaluating outcome
Justinger et al. [27]	Single-center RCT	856 (485/371)	Laparotomy	Triclosan-coated polydioxanone	6.4% (31/485)	Uncoated polydioxanone	11.3% (42/371)	CDC criteria, clinical examination during hospital stay and 2 weeks postoperatively
Nakamura et al. [30]	Single-center RCT	577 (206/204)	Colorectal surgery (open and laparoscopic)	Triclosan-coated polyglactin 910	4.4% (9/206)	Uncoated polyglactin 910	9.3% (19/204)	CDC criteria, clinical examination during hospital stay and weekly until day 30
Diener et al. [25]	Multi-center RCT	1185 (587/598)	Midline abdominal laparotomy	Triclosan-coated polydioxanone	14.8% (87/587)	Uncoated polydioxanone	16.1% (96/598)	CDC criteria, clinical examination on day 10, day of discharge and day 30
Matavelli et al. [29]	Multi-center RCT	281 (140/141)	Colorectal surgery (open and laparoscopic)	Triclosan-coated polydioxanone	12.9% (18/140)	Uncoated polydioxanone	10.6% (15/141)	CDC criteria, weekly clinical examination for 30 days after discharge
Ruiz-Tovar et al. [33]	Multi-center RCT	101 (50/51)	Laparotomy due to faecal peritonitis	Triclosan-coated polyglactin 910	10% (5/50)	Uncoated polyglactin 910	35% (18/51)	CDC criteria, clinical examination on day 5, 30 and 60
Koujalagi et al. [28]	Single-center RCT	60 (30/30)	Open abdominal surgeries	Triclosan-coated polydioxanone	30% (9/30)	Uncoated polydioxanone	43% (13/30)	CDC criteria, clinical examination on day 2, 6 and 10
Ichida et al. [26]	Single-center RCT	1013 (508/505)	Open and laparoscopic gastroenterological surgery	Triclosan-coated polyglactin 910	6.9% (35/508)	Uncoated polyglactin 910	5.9% (30/505)	CDC criteria, clinical examination during hospital stay and day 30
Olmez et al. [32]	Single-center RCT	890 (445/445)	Laparotomy for any gastrointestinal pathology	Triclosan-coated polydioxanone	19.1% (85/445)	Uncoated polydioxanone	25.8% (115/445)	Similar criteria as CDC, Clinical examination on day 7, 14 and 30
Ruiz-Tovar et al. [34]	Multi-center RCT	92 (45/47)	Laparotomy for abdominal infection and ischemia	Triclosan-coated polydioxanone	8% (4/45)	Uncoated polydioxanone	23% (11/47)	CDC criteria, clinical examination during hospital stay and day 30
NIHR [31]	Multi-center RCT	4535 (2267/2268)	Abdominal surgery with a skin incision of 5 cm or greater	Triclosan-coated polydioxanone	20.6% (467/2267)	Uncoated sutures	21.7% (493/2268)	CDC criteria, clinical examination until day 30
Kang et al. [35]	Single-center RCT	811 (396/415)	Colorectal surgery (open and laparoscopic)	Triclosan-coated polyglactin 910	3.5% (14/396)	Uncoated polyglactin 910	6.0% (25/415)	CDC criteria, clinical examination until day 30

**Fig. 3:**
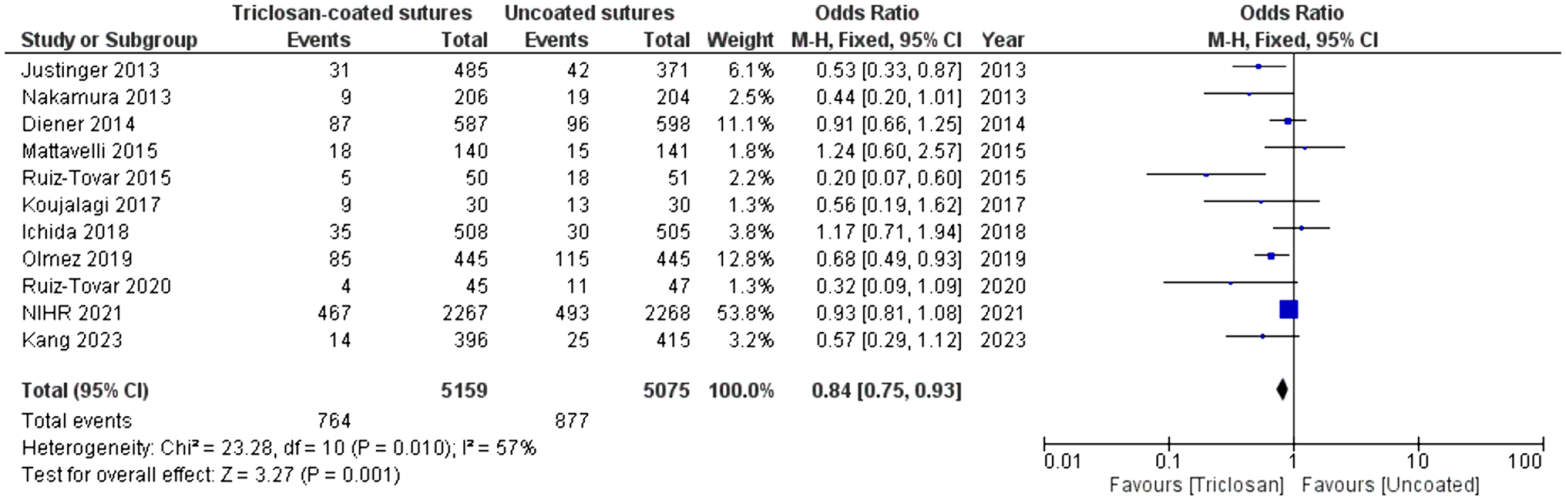
Forest plot of the OR for SSI incidence comparing triclosan-coated and uncoated sutures, created with RevMan

Only three studies reported a statistically significant difference in the SSI rate between patients treated with triclosan-coated and uncoated sutures [27, 32, 33]. According to the meta-analysis, the overall incidence of SSI was statistically significantly lower with the use of triclosan-coated sutures compared to uncoated sutures, 14.8% vs. 17.3% respectively, OR 0.84 (95% CI [0.75,0.93], *p* = 0.001), (Fig. 3). A risk difference of − 0.02 (95% CI [− 0.04, − 0.01]) was calculated. The heterogeneity was moderate (I^2^ of 57%).

**Fig. 4:**
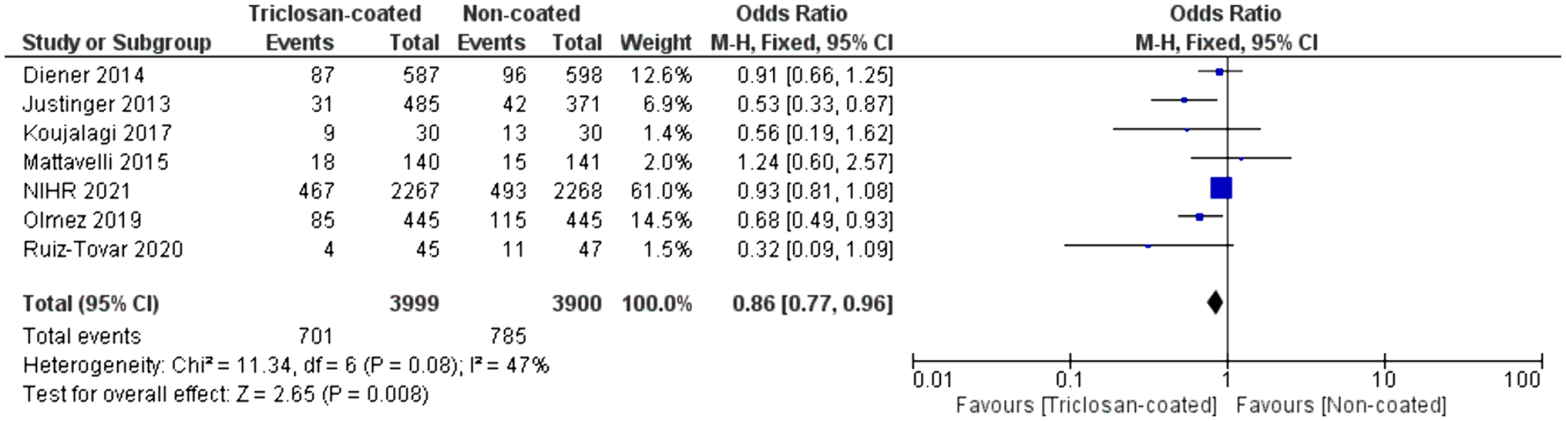
Forest plot of the OR for SSI incidence comparing triclosan-coated and uncoated polydioxanone sutures, created with RevMan

When polydioxanone sutures were evaluated separately, triclosan-coating reduced the occurrence of SSI compared to uncoated polydioxanone; 17.5% vs. 20.1%, OR 0.86, (95% CI [0.77, 0.96], *p* = 0.008), (Fig. 4).

**Fig. 5:**
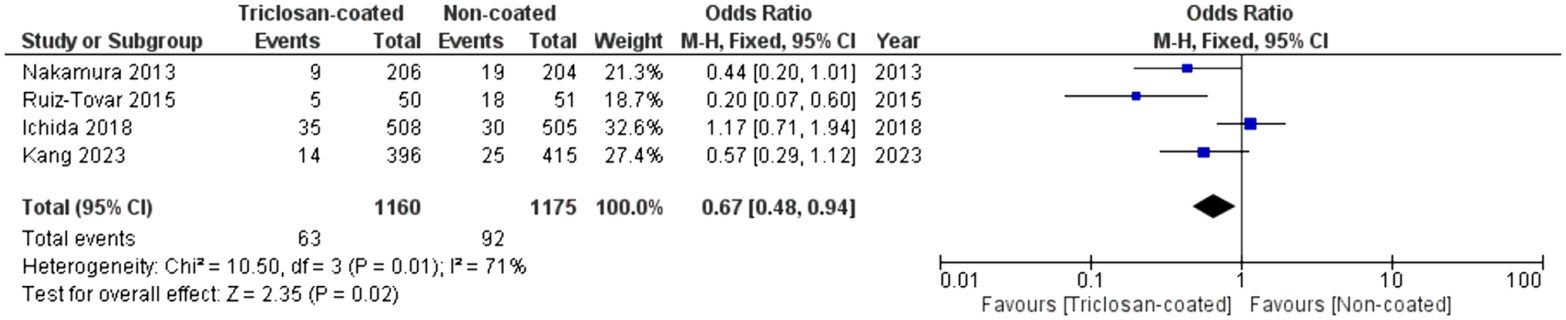
Forest plot of the OR for SSI incidence comparing triclosan-coated and uncoated polyglactin 910 sutures, created with RevMan

When polyglactin 910 was evaluated separately, triclosan-coated sutures also reduced the occurrence of SSI compared to uncoated sutures; 5.4% vs. 7.8% respectively, OR 0.67 (95% CI [0.48, 0.94], *p* = 0.02), (Fig. 5).

**Fig. 6:**
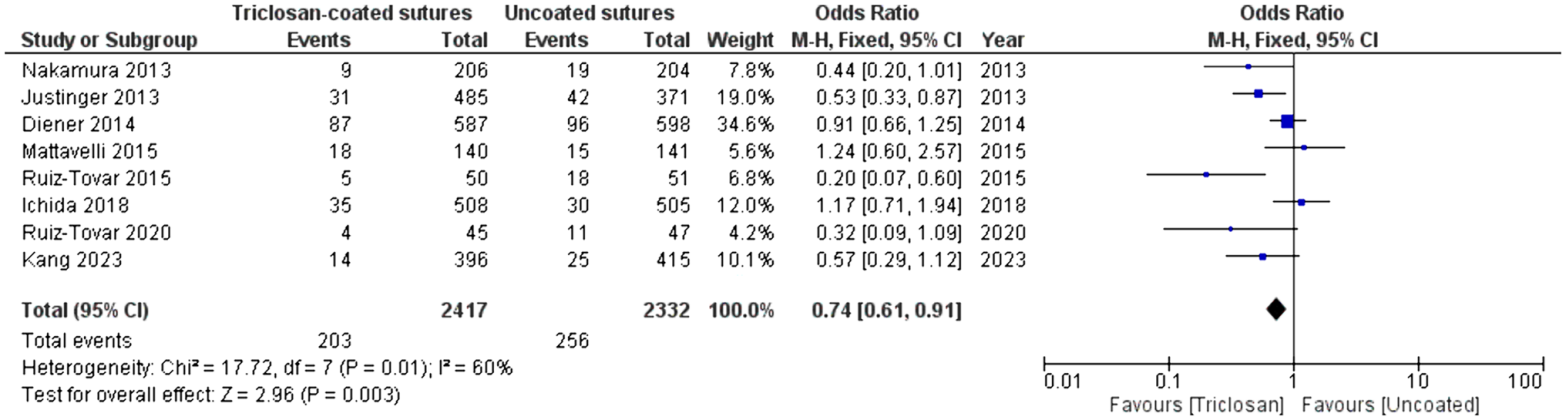
Forest plot of the OR for SSI incidence comparing triclosan-coated and uncoated sutures in high-income countries, created with RevMan

A subgroup analysis was performed including all high-income countries. The overall incidence of SSI was 9.7%. Note that the overall incidence in low- and middle-income countries was 21.5%. In the high-income countries, the SSI incidence of 8.4% was lower in the triclosan-coated suture group compared to 11.1% in the uncoated suture group, OR 0.74 (95% CI = [0.61, 0.91], *p* = 0.003), (Fig. 6). Subgroup analysis revealed that in the polydioxanone group, the overall SSI rate was 12.6%, with 11.1% in the coated group and 14.2% in the uncoated group. The OR was 0.79 (95% CI = [0.62, 1.00], *p* = 0.08) favouring triclosan-coated polydioxanone.

Sensitivity analysis showed that the significance of all the results was not sensitive to changing the endpoint to relative ratio or risk difference, to changing between random and fixed effects or to exclusion of outliers or studies creating heterogeneity. Additionally, the exclusion of articles with a risk of bias of ‘some concerns’, did not significantly change the results. Ruiz-Tovar et al. [33] was not within the 95% confidence interval lines in the funnel plot. There was some asymmetry in the funnel plot because of three studies [28, 33, 34], suggesting an overestimation of the intervention. When performing a sensitivity analysis by excluding these studies, no difference in significance was found.

## Discussion

This meta-analysis, which included eleven RCTs with 10,234 patients, revealed that there was a statistically significant risk difference of about 2% in the incidence of SSIs after abdominal surgery when suturing the fascia with triclosan-coated sutures compared to uncoated sutures.

The overall incidence of SSI after abdominal surgery was 16.0%. In three of the eleven included RCTs, the SSI incidence in the intervention group and the control group was above the average ranging from 19 to 43% as shown in Table 2 [28, 31, 32] with an overall rate of 21.5%. Notably, all of these RCTs were performed in low- and middle-income countries. It is known that the incidence of SSI is greater in low-income countries [36]. Therefore, we conducted a subgroup analysis using only high-income countries. In this subgroup the incidence of SSI ranged from 6.8 to 16.3%. Only Ruiz-Tovar et al. [33] reported an incidence of 22.8%, probably due to the exclusive inclusion of patients who underwent a laparotomy for fecal peritonitis. According to the subgroup analysis of high-income countries, the effect of the triclosan-coated sutures was greater. In the polydioxanone group, the effect was not statistically significant.

Koujalagi et al. [28] sticks out in terms of SSI rates, namely 30% and 43.3% in the intervention and control groups, respectively. This study included a total of sixty patients and was performed in India, a low-income country. In addition, follow-up was short. The surgical wound was assessed last on postoperative day ten, but postoperative wound infections can still develop up to thirty days after surgery [2]. The same applies to the study by Justinger et. al [27], in which the postoperative wound was last assessed on postoperative day fourteen. Nevertheless, these two RCTs [27, 28] together accounted for less than ten percent of the population included in this meta-analysis. All other studies had a follow-up of at least thirty days.

Overall, there was a difference in the effects of the triclosan-coating on polydioxanone sutures and polyglactin 910 sutures. This difference is difficult to explain. A meta-analysis by Patel et al. [37] with more than 6500 patients revealed no difference in the incidence of SSI after laparotomy when monofilament sutures, such as polydioxanone, were compared to multifilament sutures such as polyglactin 910 [37]. Perhaps the duration of suture absorption is a factor in whether an SSI develops. Another possibility is that there is more triclosan coating on a braided suture. In any case, SSI is a multifactorial problem for which follow-up research seems warranted.

Recently, a systematic review and meta-analysis were published by the National Institute of Health Research (NIHR) Unit on Global Surgery [38] which concluded that there was no statistically significant difference in the incidence of SSI between the triclosan-coated group and the uncoated group. However, this systematic review excluded several RCTs based on an unvalidated quality assessment tool developed by the study team itself. For example, only studies that used the validated CDC criteria for diagnosing an SSI were included in the systematic review of the NIHR. This could also lead to bias, as alternative definitions of SSI may not provide the same information. In our systematic review, several of the RCTs excluded by the NIHR review met the inclusion criteria, which resulted in a larger sample size and a possibly better summary of the available evidence on triclosan sutures. Another debatable point is that nearly two-thirds of the patients analyzed in the NIHR meta-analysis belonged to the FALCON trial [31], which took place in low- or middle-income countries with potentially inadequate infection prevention, independent of the type of suture used.

Ahmed et al. [39] published a systematic review with a large number of included articles comparing triclosan-coated and uncoated sutures. They also reported a significantly reduced risk of SSIs. Our review included four more recent studies [31, 32, 34, 35]. An important difference in our review, is that we excluded all studies with a high risk of bias. Edwards et al. [40] published a systematic review including all possible surgeries including dental and orthopedic surgeries. This meta-analysis also revealed a significantly reduced risk of SSI in the triclosan-coated suture group. Studies with a high risk of bias were not excluded. Our systematic review focused on abdominal surgery, because bacterial characteristics are expected to differ between abdominal and dental surgery for example. The expected risk reduction seems lower in this review than in all previously mentioned systematic reviews. This could be due to the characteristics of the surgery and the incision. Additionally, in our systematic review, there seems to be a lower risk of bias.

In the majority of SSIs, skin bacteria (such as *Staphylococcus aureus* and *Staphylococcus epidermis*) are the causative agents [41]. There are insufficient data available to calculate differences in bacteria, such as triclosan-sensitive species. An additional reduction in SSIs might be observed when the fascia and skin are both closed with triclosan-coated sutures. Future studies investigating this factor could provide new insights. All but three studies used no subcutaneous closure and skin staples for skin closure. Olmez et al. [32] used uncoated polypropylene sutures in all patients. Interestingly, two studies [26, 29] used uncoated and coated absorbable sutures for skin closure, depending on the coating of the sutures of the fascia. These studies showed no significant difference in the SSI rate, but no conclusions can be drawn.

This study is an updated version of the earlier TRISTAN systematic review [16]. In this update, the Cochrane RoB 2 tool was used to assess the risk of bias instead of the Scottish Intercollegiate Guidelines Network checklists. This led to the exclusion of three articles in this update due to the lack of blinding or missing description of the criteria and of the observer for the primary outcome.

There are some limitations in our study. In NIHR et al. [31], hospitals could choose to use polyglactin 910 or polydioxanone in the control group. Additionally, no distinction could be made in our meta-analysis between elective or emergency surgery. Patients undergoing emergency surgery may be more likely to have a contaminated abdomen, which may override the effect of the triclosan-coated sutures because of a greater chance of SSI in a contaminated condition. Furthermore, there was not enough data to make statements about cost-efficiency. Future studies should investigate this additional factor.

## Conclusion

This review and meta-analysis of RCTs comparing triclosan-coated to uncoated sutures for abdominal fascia closure, a statistically significant decrease in SSI with a risk difference of about 2% was found when triclosan-coated sutures were used.

### Supplementary Information

Below is the link to the electronic supplementary material.

Supplementary file1 (DOCX 20 KB)

Supplementary file2 (DOCX 17 KB)

## Data Availability

All necessary data is already given in the review and the supplementary material. When readers would like specific data, they can always send the corresponding author.
