# Supplementary material for: Systematic review and meta-analysis comparing surgical site infection in abdominal surgery between triclosan-coated and uncoated sutures
Source: Hernia. 2024 May 7;28(4):1017–27. doi: 10.1007/s10029-024-03045-5 (PMC11297069; doi:10.1007/s10029-024-03045-5)
Supplement: Supplementary file 1 — Supplementary file1 (DOCX 20 KB) [file 10029_2024_3045_MOESM1_ESM.docx]

Systematic review and meta-analysis comparing surgical site infection in abdominal surgery between Triclosan‐coated and non-coated sutures.

Date: 17.02.2024

| **Database searched** | **Platform** | **Years of coverage** | **Records** | **Records after duplicates removed** |
| --- | --- | --- | --- | --- |
| Embase | Embase.com | 1971 - Present | 551 | 545 |
| Medline ALL | Ovid | 1946 - Present | 220 | 218 |
| Web of Science Core Collection* | Web of Knowledge | 1975 - Present | 358 | 356 |
| Cochrane Central Register of Controlled Trials | Wiley | 1992 - Present | 394 | 382 |
| **Total** | | | **1523** | **1501** |
| **Total after duplicates removed** | | | **-** | **1290** |

*Science Citation Index Expanded (1975-present) ; Social Sciences Citation Index (1975-present) ; Arts & Humanities Citation Index (1975-present) ; Conference Proceedings Citation Index- Science (1990-present) ; Conference Proceedings Citation Index- Social Science & Humanities (1990-present) ; Emerging Sources Citation Index (2015-present)

**Embase – 551**

('abdominal surgery'/de OR 'laparotomy'/de OR (laparotom* OR celiotom* OR peritoneotom* OR ((abdom*) NEAR/3 (surg* OR operation OR closur*))):ab,ti,kw) AND ('suture'/exp OR (PDS OR sutur* OR Vicryl* OR Monomax OR monocryl* OR petcryl* OR Mitsu* OR polyglactin):ab,ti,kw) AND ('surgical infection'/de OR 'wound infection'/de OR (SSI OR SSO OR ((surg* OR wound* OR postoperat*) NEAR/3 (infect* OR occurrenc* OR contaminat* OR sepsis*))):ab,ti,kw) **AND** ('Controlled clinical trial'/exp OR 'Crossover procedure'/de OR 'Double-blind procedure'/de OR 'Single-blind procedure'/de OR (random* OR factorial* OR crossover* OR (cross NEXT/1 over*) OR placebo* OR ((doubl* OR singl*) NEXT/1 blind*) OR assign* OR allocat* OR volunteer* OR trial OR groups):ab,ti,kw)

**Medline – 220**

(Laparotomy/ OR (laparotom* OR celiotom* OR peritoneotom* OR ((abdom*) ADJ3 (surg* OR operation OR closur*))).ab,ti,kf.) AND (Sutures/ OR (PDS OR sutur* OR Vicryl* OR Monomax OR monocryl* OR petcryl* OR Mitsu* OR polyglactin).ab,ti,kf.) AND (surgical infection/ OR wound infection/ OR (SSI OR SSO OR ((surg* OR wound* OR postoperat*) ADJ3 (infect* OR occurrenc* OR contaminat* OR sepsis*))).ab,ti,kf.) AND (Exp Controlled clinical trial/ OR "Double-Blind Method"/ OR "Single-Blind Method"/ OR "Random Allocation"/ OR (random* OR factorial* OR crossover* OR cross over* OR placebo* OR ((doubl* OR singl*) ADJ blind*) OR assign* OR allocat* OR volunteer* OR trial OR groups).ab,ti,kf.) NOT (exp Animals/ NOT Humans/)

**Web of Science – 358**

TS=(((laparotom* OR celiotom* OR peritoneotom* OR ((abdom*) NEAR/2 (surg* OR operation OR closur*)))) AND ((PDS OR sutur* OR Vicryl* OR Monomax OR monocryl* OR petcryl* OR Mitsu* OR polyglactin)) AND ((SSI OR SSO OR ((surg* OR wound* OR postoperat*) NEAR/2 (infect* OR occurrenc* OR contaminat* OR sepsis*)))) AND ((random* OR factorial* OR crossover* OR (cross NEAR/1 over*) OR placebo* OR ((doubl* OR singl*) NEAR/1 blind*) OR assign* OR allocat* OR volunteer* OR trial OR groups)))

**Cochrane – 394**

((laparotom* OR celiotom* OR peritoneotom* OR ((abdom*) NEAR/3 (surg* OR operation OR closur*))):ab,ti,kw) AND ((PDS OR sutur* OR Vicryl* OR Monomax OR monocryl* OR petcryl* OR Mitsu* OR polyglactin):ab,ti,kw) AND ((SSI OR SSO OR ((surg* OR wound* OR postoperat*) NEAR/3 (infect* OR occurrenc* OR contaminat* OR sepsis*))):ab,ti,kw)
