# Supplementary material for: Systematic review and meta-analysis comparing surgical site infection in abdominal surgery between triclosan-coated and uncoated sutures
Source: Hernia. 2024 May 7;28(4):1017–27. doi: 10.1007/s10029-024-03045-5 (PMC11297069; doi:10.1007/s10029-024-03045-5)
Supplement: Supplementary file 2 — Supplementary file2 (DOCX 17 KB) [file 10029_2024_3045_MOESM2_ESM.docx]

1. Abdeldaim HM, Ahmed TAF, Alsyiedand EGM, Zaitoun MM. Wound complications in obese patients after major gynecologic oncology surgeries, an intervention study. Turkish journal of physiotherapy and rehabilitation. 2021;32(3):12434‐40.

2. Ademuyiwa AO, Hardy P, Runigamugabo E, et al. Reducing surgical site infections in low-income and middle-income countries (FALCON): a pragmatic, multicentre, stratified, randomised controlled trial. The Lancet. 2021;398(10312):1687-99.

3. Baracs J, Huszár O, Sajjadi SG, Peter Horváth O. Surgical site infections after abdominal closure in colorectal surgery using triclosan-coated absorbable suture (PDS Plus) vs. Uncoated sutures (PDS II): A randomized multicenter study. Surgical Infections. 2011;12(6):483-9.

4. Cózar Lozano C, Garcia-Botello S, Martí-Arévalo J, et al. Use of triclosan-coated barbed monofilament suture (TCBMS) to reduce surgical site infection (SSI) in elective colorectal surgery. Diseases of the Colon and Rectum. 2020;63(6):e441.

5. Ford HR, Jones P, Gaines B, et al. Intraoperative handling and wound healing: controlled clinical trial comparing coated VICRYL plus antibacterial suture (coated polyglactin 910 suture with triclosan) with coated VICRYL suture (coated polyglactin 910 suture). Surgical infections. 2005;6(3):313-21.

6. Galal I, El-Hindawy K. Impact of using triclosan-antibacterial sutures on incidence of surgical site infection. American journal of surgery. 2011;202(2):133-8.

7. Golub AV, Kozlov RS, Pleshkov VG, et al. Surgical Site Infections after Open Appendectomy and Effectiveness of Complex Approach to Their Prevention. Khirurgiia. 2016(6):68‐76.

8. Heger P, Pianka F, Diener MK, Mihaljevic AL. Current standards of abdominal wall closure techniques. Conventional suture techniques. Chirurg. 2016;87(9):737-43.

9. Heger U, Voss S, Knebel P, et al. Prevention of abdominal wound infection (PROUD trial, DRKS00000390): study protocol for a randomized controlled trial. Trials. 2011;12:10.

10. Henriksen NA, Deerenberg EB, Venclauskas L, et al. Triclosan-coated sutures and surgical site infection in abdominal surgery: the TRISTAN review, meta-analysis and trial sequential analysis. Hernia. 2017;21(6):833-41.

11. Hoshino S, Yoshida Y, Tanimura S, et al. A study of the efficacy of antibacterial sutures for surgical site infection: a retrospective controlled trial. Int Surg. 2013;98(2):129-32.

12. Huszár O, Baracs J, Tóth M, et al. Comparison of wound infection rates after colon and rectal surgeries using triclosan-coated or bare sutures -- a multi-center, randomized clinical study. Magyar sebeszet. 2012;65(3):83‐91.

13. Jprn U. Triclosan Coated Sutures for the Reduction of Abdominal Wound Infections and Economic Considerations :Single institutional prospective randomized control trial. <https://trialsearchwhoint/Trial2aspx?TrialID=JPRN-UMIN000003322>. 2010.

14. Jprn U. Does antimicrobial triclosan-coated PDS PLUS for skin closure reduce surgical site infections? A controlled clinical trial of class II abdominal surgeries. <https://trialsearchwhoint/Trial2aspx?TrialID=JPRN-UMIN000021892>. 2016.

15. Justinger C, Moussavian MR, Schlueter C, et al. Antibacterial [corrected] coating of abdominal closure sutures and wound infection. Surgery. 2009;145(3):330-4.

16. Justinger C, Schuld J, Sperling J, et al. Triclosan-coated sutures reduce wound infections after hepatobiliary surgery--a prospective non-randomized clinical pathway driven study. Langenbecks Arch Surg. 2011;396(6):845-50.

17. Justinger C, Slotta JE, Schilling MK. Incisional hernia after abdominal closure with slowly absorbable versus fast absorbable, antibacterial-coated sutures. Surgery. 2012;151(3):398-403.

18. Khachatryan N, Dibirov M, Omelyanovsky V, et al. Prevention of postoperative infections in abdominal surgery using reabsorbable suture with antibacterial activity (Vicryl Plus) versus reabsorbable standard sutures. Surgical infections. 2011;12(2):A13‐4.

19. Khan R, Yee AL, Gilbert JA, et al. Triclosan-containing sutures: safety and resistance issues need to be addressed prior to generalized use. Appl Nanosci.10.

20. Konstantelias AA, Andriakopoulou CSI, Mourgela S. Triclosan-coated sutures for the prevention of surgical-site infections: a meta-analysis. Acta Chirurgica Belgica. 2017;117(3):137-48.

21. Leaper DJ, Edmiston CE, Holy CE. Meta-analysis of the potential economic impact following introduction of absorbable antimicrobial sutures. British Journal of Surgery. 2017;104(2):E134-E44.

22. Lee CY, Jacob VA, Premchandran N. Triclosan impregnated suture in management of acute surgical wounds: A review of current evidences. Malaysian Orthopaedic Journal. 2018;12.

23. Li D, Zhuang J, Liu YG, et al. Full fascia closure with interrupted absorbable suture and layered closure with interrupted silk suture in abdominal incision: comparison of curative effects and biocompatibility. Chinese journal of tissue engineering research. 2014;18(43):6996‐7000.

24. Mankin KMT, Cohen ND. Randomized, controlled clinical trial to assess the effect of antimicrobial-impregnated suture on the incidence of surgical site infections in dogs and cats. JAVMA-J Am Vet Med Assoc. 2020;257(1):62-9.

25. Mattavelli I, Nespoli L, Alfieri S, et al. Effect of triclosan-coated suture on surgical site infection after colorectal surgery: final results of a multicenter, prospective, randomized trial. Surgical infections. 2013;14(2):A9.

26. Mattavelli I, Nespoli L, Alfieri S, et al. Triclosan-coated suture to reduce surgical site infection after colorectal surgery. Surgical Infections. 2011;12(2):A14-A5.

27. Mingmalairak C, Ungbhakorn P, Paocharoen V. Efficacy of antimicrobial coating suture coated polyglactin 910 with tricosan (Vicryl plus) compared with polyglactin 910 (Vicryl) in reduced surgical site infection of appendicitis, double blind randomized control trial, preliminary safety report. Chotmaihet thangphaet [Journal of the Medical Association of Thailand]. 2009;92(6):770‐5.

28. Miyoshi N, Fujino S, Nishimura J, et al. Effectiveness of Triclosan-Coated Sutures Compared with Uncoated Sutures in Preventing Surgical Site Infection after Abdominal Wall Closure in Open/Laparoscopic Colorectal Surgery. Journal of the American College of Surgeons. 2022;234(6):1147-59.

29. Nakamura T, Sato T, Takayama Y, et al. Risk Factors for Surgical Site Infection after Laparoscopic Surgery for Colon Cancer. Surgical Infections. 2016;17(4):454-8.

30. Nct. PDS*Plus and Wound Infections After Laparotomy. <https://clinicaltrialsgov/show/NCT00998907>. 2009.

31. Nct. Antiseptic Sutures and Wound Infection. <https://clinicaltrialsgov/show/NCT00932503>. 2009.

32. Nct. Abdominal Wall Closure With Triclosan-coated Suture (TCS09-10). <https://clinicaltrialsgov/show/NCT01123616>. 2010.

33. Nct. Clinical Outcome in View of Surgical Site Infection (SSI) With Antibacterial Skin Sutures. <https://clinicaltrialsgov/show/NCT01540279>. 2012.

34. Nct. Impact of Triclosan-coated Suture on Surgical Site Infection After Colorectal Surgery. <https://clinicaltrialsgov/show/NCT01869257>. 2013.

35. Nct. FALCON Trial Testing Measures to Reduce Surgical Site Infection. <https://clinicaltrialsgov/show/NCT03700749>. 2018.

36. Nct. Effect of Barbed Suture and Triclosan-coated Monofilament in Emergency Surgery. <https://clinicaltrialsgov/show/NCT03763279>. 2018.

37. Okada N, Nakamura T, Ambo Y, et al. Triclosan-coated abdominal closure sutures reduce the incidence of surgical site infections after pancreaticoduodenectomy. Surgical infections. 2014;15(3):305‐9.

38. Olmez T, Colak T. The effect of triclosan coated suture material on surgical site infection of abdominal facial closure. European Surgical Research. 2015;55:66-7.

39. Rasić Z, Schwarz D, Adam VN, et al. Efficacy of antimicrobial triclosan-coated polyglactin 910 (Vicryl* Plus) suture for closure of the abdominal wall after colorectal surgery. Collegium antropologicum. 2011;35(2):439‐43.

40. Renko M, Paalanne N, Tapiainen T, et al. Triclosan-containing sutures versus ordinary sutures for reducing surgical site infections in children: a double-blind, randomised controlled trial. Lancet Infect Dis. 2017;17(1):50-7.

41. Roy PK, Kalita P, Lalhlenmawia H, et al. COMPARISON OF SURGICAL SITE INFECTION RATE BETWEEN ANTIBACTERIAL COATED SURGICAL SUTURE AND CONVENTIONAL SUTURE: A RANDOMIZED CONTROLLED SINGLE CENTRE STUDY FOR PREVENTIVE MEASURE OF POSTOPERATIVE INFECTION. International Journal of Pharmaceutical Sciences and Research. 2019:2385.
